# Diagnostic value of core needle biopsy for determining HER2 status in breast cancer, especially in the HER2-low population

**DOI:** 10.1007/s10549-022-06781-3

**Published:** 2022-11-08

**Authors:** Ruixian Chen, Yana Qi, Ya Huang, Weijing Liu, Ruoning Yang, Xin Zhao, Yunhao Wu, Qintong Li, Zhu Wang, Xin Sun, Bing Wei, Jie Chen

**Affiliations:** 1 Breast Center, West China Hospital, Sichuan University, No. 37 Guo Xue Alley, Chengdu, 610041 Sichuan China; 2 Chinese Evidence-Based Medicine Center, West China Hospital, Sichuan University, No. 37 Guo Xue Xiang, Chengdu, 610041 Sichuan China; 3 Departments of Obstetrics & Gynecology and Pediatrics, West China Second University Hospital, Key Laboratory of Birth Defects and Related Diseases of Women and Children, Ministry of Education, State Key Laboratory of Biotherapy and Collaborative Innovation Center of Biotherapy, Sichuan University, Chengdu, China; 4 Laboratory of Molecular Diagnosis of Cancer, West China Hospital, Sichuan University, Chengdu, China; 5 Department of Pathology, West China Hospital, Sichuan University, No. 37 Guo Xue Xiang, Chengdu, 610041 Sichuan China

**Keywords:** Breast cancer, HER2 low, Core needle biopsy, Surgical excision biopsy, Clinicopathological features, Immunohistochemistry

## Abstract

**Purpose:**

The status of human epidermal growth factor receptor 2 (HER2) is important for treatment decision-making of breast cancer and was commonly determined by core needle biopsy (CNB). The concordance of CNB with surgical excision biopsy (SEB) has been verified, but remain unclear according to the newly developed classification of HER2 status. Our study aimed to re-evaluate the diagnostic value of CNB for determining HER2 status in breast cancer, especially in the HER2-low population.

**Methods:**

Eligible breast cancer patients in West China Hospital between January 1, 2007 and December 31, 2021 were enrolled consecutively and data were extracted from the Hospital Information System. The agreement of HER2 status between CNB and SEB was calculated by concordance rate and κ statistics, as well as the sensitivity, specificity, positive, and negative predictive values (PPV & NPV). Logistic models were used to explore potential factors associated with the discordance between both tests.

**Results:**

Of 1829 eligible patients, 1097 (60.0%) and 1358 (74.2%) were consistent between CNB and SEB by pathological and clinical classifications, respectively, with κ value being 0.46 (0.43–0.49) and 0.57 (0.53–0.60). The sensitivity (50.9%–52.7%) and PPV (50.5%-55.2%) of CNB were especially low among IHC 1+ and 2+/ISH - subgroups by pathological classifications; however, it showed the highest sensitivity (77.5%) and the lowest specificity (73.9%) in HER2-low population by clinical classifications. Advanced N stages might be a stable indicator for the discordance between both tests.

**Conclusion:**

The diagnostic value of CNB was limited for determining HER2 status in breast cancer, especially in HER2-low population.

## Introduction

Human epidermal growth factor receptor 2 (HER2), which determines histopathological molecular subtypes along with estrogen receptor (ER) and progesterone receptor (PR), is an important indicator for the treatment and prognosis of breast cancer. The HER2 status is mainly assessed using immunohistochemistry (IHC) [1] and was divided into HER2 negative (IHC score 0 and 1+), HER2 equivocal (IHC score 2+), and HER2 positive (IHC score 3+) [2]. In situ hybridization (ISH) is used further for HER2-equivocal cases; tumors that are ISH positive and negative are classified as HER2 positive and negative, respectively [3].

However, in recent years, this traditional classification of HER2 status has been challenged and from a new perspective, the concept of HER2-low (IHC 1+ or 2+/ISH -) tumors was proposed considering this new subtype may possess distinct features [4]. Existing population-based study evidences showed that, comparing to HER2-negative patients, patients with HER2-low tumors would have better overall and disease-free survival outcomes [5], as well as lower pathological complete response [6]. Besides, one comparative analysis of signal pathway mutations derived from second-generation sequencing (NGS) of gene panels among breast cancer patients and demonstrated that there might be more gene mutations in the PI3K-Akt signaling pathway of HER2-low tumors than HER2-positive and HER2-negative tumors [7].

Based on HER2 status, the treatment plan including whether and how to receive anti-HER2 therapy would be developed. For a long time, anti-HER2 therapy, such as dual blockade with trastuzumab+pertuzumab and TDM-1, is only prescribed for HER2-positive breast cancer patients [8], but latest research showed that trastuzumab deruxtecan (T-DXd) shall also benefit patients with HER2-low breast cancer [9, 10], which has been validated by one multicenter, randomized controlled trial (RCT) enrolling 557 patients with HER2-low breast cancer (DESTINY-Breast 04), presenting patients treated by T-DXd had significantly longer progression-free and overall survival time than those by only chemotherapy treatment [11]. This suggested that how to distinguish HER2-low breast cancer patients from HER2-positive or -negative patients is essential for decision-making regarding whether to receive anti-HER2 treatment and selecting appropriate types of anti-HER2 agents, e.g., T-DXd prescribed for HER2-low patients.

Currently, the HER2 status is mainly determined by detecting specimens from core needle biopsy (CNB) and surgical excision biopsy (SEB) [12]. Although SEB is always regarded as gold standards, CNB is more convenient, cost-effective, and is the only alternative detection method nowadays for patients with advanced cancer or those before neoadjuvant treatment, making it become a conventional procedure in many countries [13]. Till now, there has been several studies investigating the concordance between CNB and SEB, but the concordance rate of HER2 status is ambiguous, varying from 56 to 98.3% [14–17] and those potential factors influencing the agreement of both tests remain unclear.

In view of the development regarding new classification of HER2 status, the comparative effectiveness of HER2-low breast cancer patients from treatment by T-DXd, and the outstanding value of CNB among patients with advanced cancer or those before neoadjuvant therapy, it is urgent for us to further verify the concordance of CNB and SEB, especially to evaluate the diagnostic value of CNB among patients with HER2-low breast cancer. To address all these questions, we conducted this retrospective study based on medical records of female breast cancer from one general referral hospital in southwest China, based on which, we also investigated the epidemiological characteristics of HER2-low tumors comparing with those with positive and negative HER2 expression and explored the possible influential factors that might be associated with the discordance between CNB and SEB.

## Methods

### Design and patients

This retrospective study was conducted at Breast Center in West China Hospital in Sichuan province, China. All eligible patients diagnosed with breast cancer between January 1, 2007 and December 31, 2021 were enrolled consecutively and medical records of included patients were identified from the Hospital Information System (HIS) database. The data we collected were mainly necessary demographical, clinical, and pathological characteristics, including age, tumor location, histological type, grade, stage (T & N), surgery type, ER, and PR status. The study was approved by the institutional review board in West China Hospital (No. 2022 [671]) and exempt of patient informed consent.

Eligible patients should meet all the following criteria: female; diagnosed with invasive breast cancer; and underwent both CNB and SEB, with both specimens processed by IHC staining to determine HER2 status. Patients were excluded if they met any of the following criteria: received neoadjuvant systemic therapy (NAST); received ipsilateral breast/chest radiotherapy before operation; or IHC 2+ tumors with a gene amplification reported by fluorescence in situ hybridization (FISH) only in CNB or SEB. The reason for excluding patients receiving NAST is, compared to the previous CNB, the tumor tends to present size and pathological changes on SEB resulting from neoadjuvant treatment [18, 19].

### Specimen processing

CNBs were obtained with ultrasound guided 14- or 16-gauge core needle. Conventionally, 4-6 samples of the same patient would be acquired (from center to peripheral sections of the lesion) and then fixed in 4% neutral formaldehyde for 8-12 h. SEB were obtained following lumpectomy or mastectomy, first cut tumor samples (removed during the surgery) into 5-mm sections, and last fixation (4% neutral formaldehyde) for 8-24 h. Later, tissue samples were embedded into paraffin and cut into 4-μm-thick sections (Leica RM 2245) prior to analysis. Routinely, Hematoxylin–eosin (HE) staining was performed on each section. IHC analysis were performed with specific antibody (Ventana 4B5) against HER2. The Multimer-Technology based ultraView universal DAB detection kit (Ventana) was used for IHC staining of slides prepared from formalin fixed paraffin embedded tissue on BenchMark Ultra (Ventana), an IHC automatic dyeing system. For each slide, the automated Benchmark system would put it through a series of user-defined de-paraffinization and antigen retrieval steps before commencing with the antibody staining. The primary antibody was either applied automatically in a pre-diluted dispenser or otherwise manually titrated onto the slide. Then the pre-diluted dispensers of the ultraView Universal DAB Detection Kit provided all reagents required for staining, and the staining was proceeding in the BenchMark Ultra system automatically.

### IHC test scoring

Slides from the original specimens were reviewed. The evaluation of HER2 status by IHC test scoring were independently performed by two professors with at least 10-year abundant academic and clinical experience in breast pathology. The consistency rate of evaluation results was about 95%, and any inconsistency will be resolved through consensus on a multi-head microscope. The IHC scoring of HER2 status ranges from 0 to 3+ based on membranous staining [3]. 0 refers to no staining or weak membrane staining in < 10% of tumor cells, while incomplete or weak/barely visible membrane staining in ≥ 10% of tumor cells is 1+. 2+ means weak to moderate complete membrane staining in > 10% of tumor cells. Strong intact membrane staining observed in > 10% of tumor cells is regarded as 3+. For sections scoring 2+ in CNBs and SEBs, FISH would be applied for further pathological confirmation. When HER2 gene amplification was reported by FISH, the HER2 status was regarded as positive [3]. IHC 1+ and IHC 2+/FISH - cases were categorized into HER2-low tumors [4]. Since heterogeneity might exist in the results of FISH [20], we cannot ensure the precise status of HER2 when gene amplification was reported by FISH only in CNB or SEB, thus we excluded patients with HER2 gene amplification in only one of the specimens (CNB/SEB).

### Statistical analysis

Our dataset had no missing data across variables of our interest. We described demographical, clinical, and pathological characteristics by the HER2 status (negative, low, and positive) in SEB. The numbers and percentages were used for categorical variables, while mean and standard deviation (SD) for continuous variables. The Chi-square test or Fisher’s exact test was used to compare the differences of characteristics between patients with different HER-2 statuses.

The agreement of HER2 status by both pathological and clinical classification between CNB and SEB was measured by concordance rate and kappa (κ) statistics and determined κ values with > 0.80, 0.61-0.80, 0.41-0.6, 0.21-0.4, and < 0.2 as excellent, substantial, moderate, fair, and poor, respectively. The Sankey diagrams was used to visualize the concordance of HER2 status between both tests. The sensitivity, specificity, positive (PPV), and negative predictive values (NPV) of CNB in different pathological and clinical categories of HER2 status by SEB were calculated to comprehensively investigate the value of CNB.

To explore potential influencing factors that might result in discordance between CNB and SEB, we conducted both univariable and multivariable logistic regression analysis, by setting the consistency of CNB and SEB on pathological or clinical classification as reference of dependent variable and included different independent variables in multivariable model according to three strategies: full model, stepwise backward based on Akaike information criterion (AIC), and univariable analysis with *P* < 0.05, with the area under the ROC curve used for model evaluation. The odds ratio (OR) and 95% confidence interval (CI) of each included factor were calculated.

We conducted all the statistical analyses by SPSS 21.0 (SPSS Inc., Chicago, IL, USA) and R software (R version 4.0.4). The test of two-side *P*-value less than 0.05 was defined as statistically significant.

## Results

### Patient characteristics

**Fig. 1:**
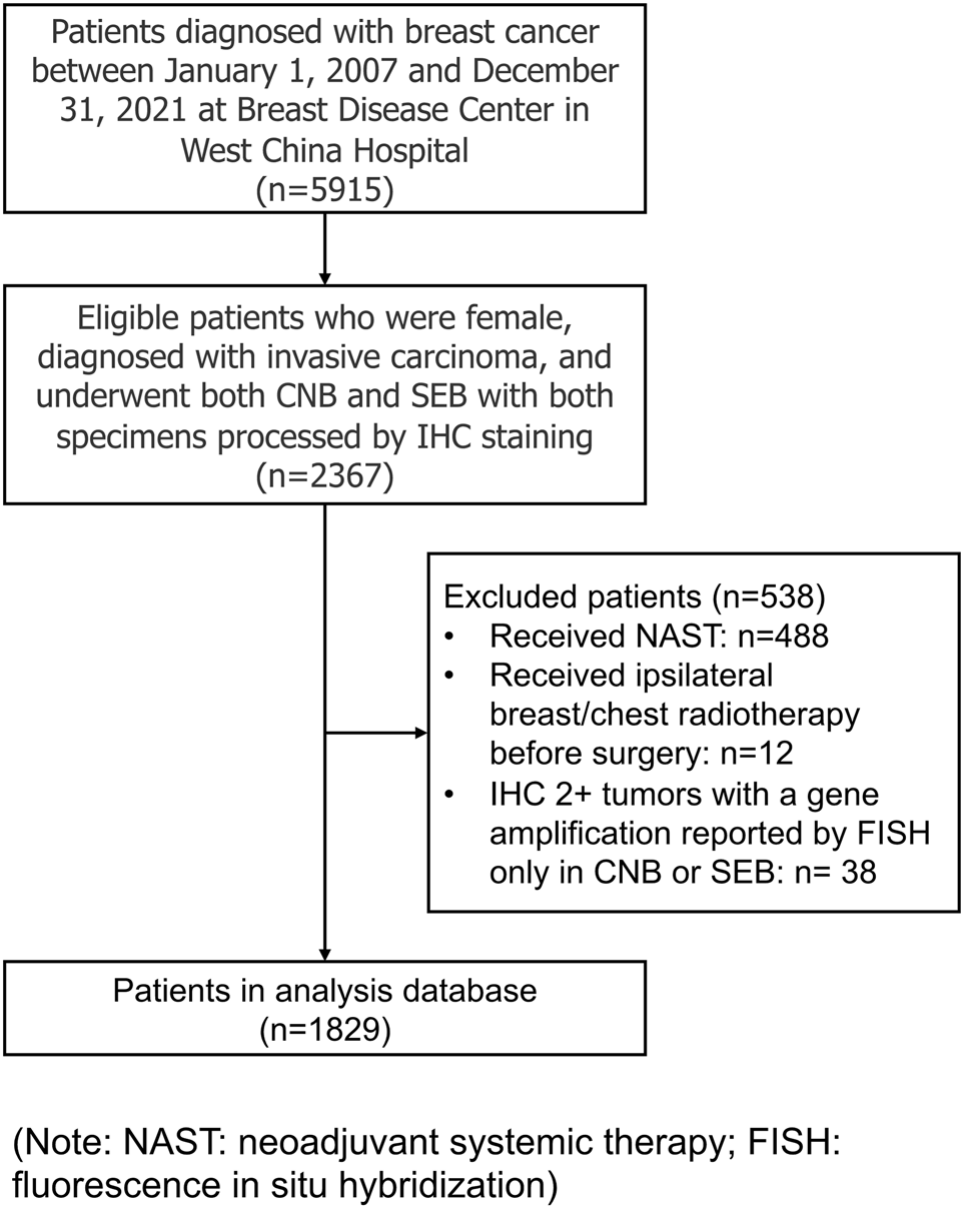
Flowchart of included population. NAST, neoadjuvant systemic therapy; FISH, fluorescence in situ hybridization

Initially, we identified 5915 breast cancer patients in target hospital and included a total of 1829 eligible patients for analysis (Fig. 1). There were 1016 (55.5%) patients diagnosed as HER2 low in SEB (IHC 1+ and 2+/ISH -), with 462 (25.3%) and 351 (19.2%) patients as HER2 negative and positive, respectively. The demographical, clinical, and pathological characteristics of patients are shown in Table 1. The average age of included patients were 51.1 years (SD: 11.5); 23 (1.3%) located in bilateral breast; 1727 (94.4%) were invasive ductal carcinoma; and histological grade 3, T3-T4 stages, and N2-N3 stages accounted for 51.3%, 9.1%, and 21.2%, respectively. 1658 (90.7%) patients underwent mastectomy surgery. 1324 (72.4%) and 1230 (67.2%) patients were diagnosed as ER positive and PR positive in SEB. Compared to those with HER2-negative or positive tumors, patients with HER2-low tumors were less inclined to be histological Grade 3 and T3-T4 stage; but more likely to be ER and PR positive; all differences were shown statistically significant with *P* < 0.05. Besides, it seemed that HER2 expression level might increase with age but remained to be verified. (Table 1).

**Table 1:** Demographical, clinical, and pathological characteristics of included patients (n, %)

Characteristics		Overall (n = 1829)	HER2 status in SEB			*p*-value*
			Negative (n = 462)	Low (n = 1016)	Positive (n = 351)	
*Age group (years)*						
≤ 35		132 (7.2)	46 (10.0)	66 (6.5)	20 (5.7)	**0.001**
36–45		497 (27.2)	137 (29.7)	276 (27.2)	84 (23.9)	
46–55		592 (32.4)	144 (31.2)	319 (31.4)	129 (36.8)	
56–65		391 (21.4)	75 (16.2)	226 (22.2)	90 (25.6)	
> 65		217 (11.9)	60 (13.0)	129 (12.7)	28 (8.0)	
*Tumor location*						
Unilateral		1806 (98.7)	453 (98.1)	1009 (99.3)	344 (98.0)	0.051
Bilateral		23 (1.3)	9 (1.9)	7 (0.7)	7 (2.0)	
Histological type						
Invasive ductal		1727 (94.4)	429 (92.9)	961 (94.6)	337 (96.0)	0.143
Others		102 (5.6)	33 (7.1)	55 (5.4)	14 (4.0)	
*Histological Grade*						
G1		63 (3.4)	22 (4.8)	38 (3.7)	3 (0.9)	** < 0.001**
G2		828 (45.3)	178 (38.5)	552 (54.3)	98 (27.9)	
G3		938 (51.3)	262 (56.7)	426 (41.9)	250 (71.2)	
*pT stage*						
T1		708 (38.7)	174 (37.7)	435 (42.8)	99 (28.2)	** < 0.001**
T2		954 (52.2)	248 (53.7)	497 (48.9)	209 (59.5)	
T3-T4		167 (9.1)	40 (8.7)	84 (8.3)	43 (12.3)	
*pN stage*						
N0		924 (50.5)	224 (48.5)	527 (51.9)	173 (49.3)	0.488
N1		517 (28.3)	131 (28.4)	289 (28.4)	97 (27.6)	
N2-N3		388 (21.2)	107 (23.2)	200 (19.7)	81 (23.1)	
*Type of surgery*						
Mastectomy		1658 (90.7)	413 (89.4)	915 (90.1)	330 (94.0)	0.050
Lumpectomy		171 (9.3)	49 (10.6)	101 (9.9)	21 (6.0)	
*ER status in SEB*						
Negative		505 (27.6)	137 (29.7)	183 (18.0)	185 (52.7)	** < 0.001**
Positive		1324 (72.4)	325 (70.3)	833 (82.0)	166 (47.3)	
*PR status in SEB*						
Negative		599 (32.8)	182 (39.4)	215 (21.2)	202 (57.5)	** < 0.001**
Positive		1230 (67.2)	280 (60.6)	801 (78.8)	149 (42.5)	

SEB, surgical excision biopsy; ER, estrogen receptor; PR, progesterone receptor

* Chi-square test

### Concordance of HER2 status between CNB and SEB

**Fig. 2:**
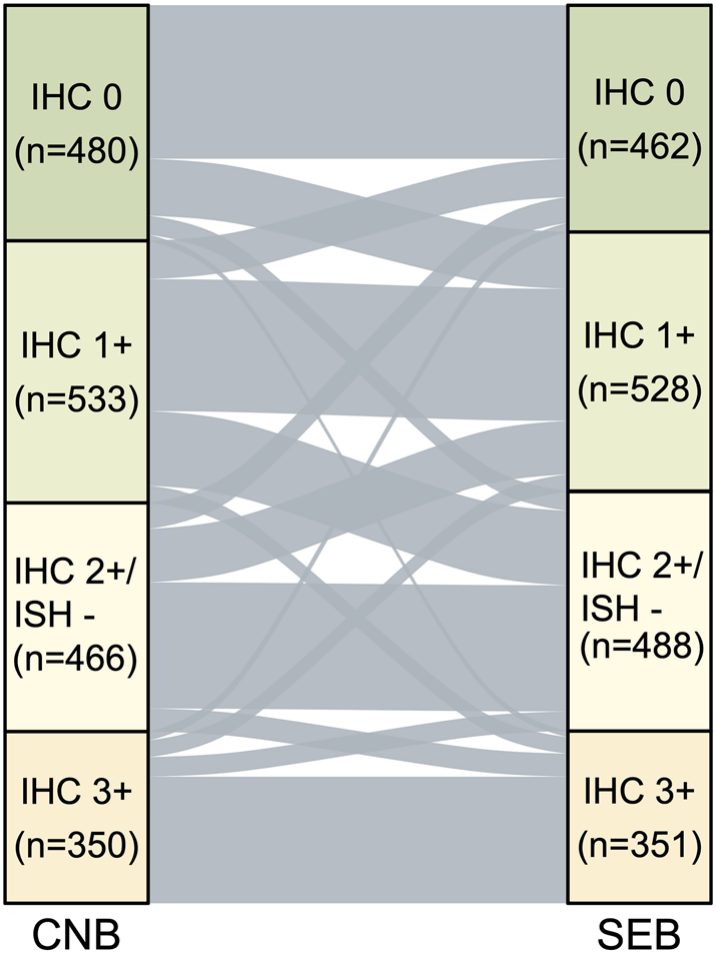
Sankey diagrams illustrating concordance of HER2 status between CNB and SEB by pathological classification. IHC, immunohistochemistry; ISH, in situ hybridization; CNB, core needle biopsy; SEB, surgical excision biopsy

**Fig. 3:**
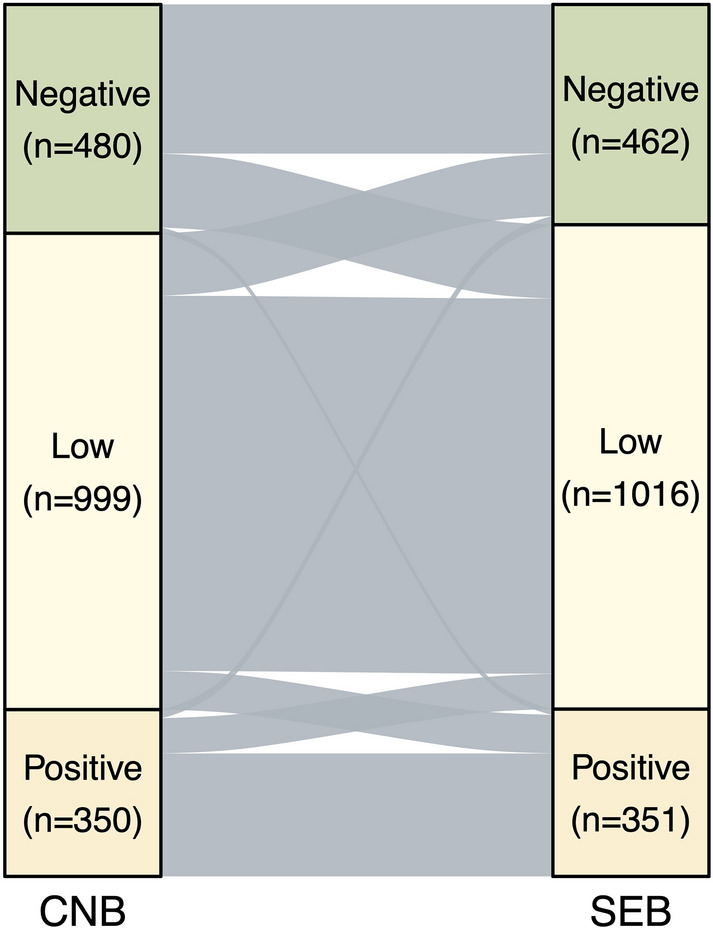
Sankey diagrams illustrating concordance of HER2 status between CNB and SEB by clinical classification. CNB, core needle biopsy; SEB, surgical excision biopsy

Figures 2 and 3 illustrate the concordance of HER2 status between CNB and SEB on pathological and clinical classifications, respectively. The concordance rate of HER2 status between CNB and SEB on pathological classification were 1097/1829 (60.0%) overall with κ being 0.46 (0.43-0.49). When we categorized IHC 1+ and IHC 2+/ISH - cases into a HER2-low group, the concordance rate (1358/1829; 74.2%) among three groups (negative/low/positive) increased compared to the initial value of four classes, with a κ value of 0.57 (0.53-0.60) indicating moderate consistency. As shown in Table 2 and Table 3, 15.3% (155/1016) and 7.3% (74/1016) HER2-low tumors would be misclassified as HER2-negative and HER2-positive (3+) tumors by CNB, respectively; meanwhile, 28.4% (131/462) HER2-negative and 23.1% (81/351) HER2-positive tumors would be misclassified as HER2-low tumors by CNB.

**Table 2:** Concordance of HER2 status between CNB and SEB by pathological classification (n, %)

Core needle biopsy (CNB)	Surgical excision biopsy (SEB)				Concordance rate (%)	κ value (95%CI)	*p-*value
	0 (n = 462)	1+ (n = 528)	2+/ISH - (n = 488)	3+ (n = 351)			
0	313 (67.7)	116 (22.0)	39 (8.0)	12 (3.4)	60.0	0.46	< 0.001
1+	78 (16.9)	269 (50.9)	152 (31.1)	34 (9.7)		(0.43-0.49)	
2+/ISH -	53 (11.5)	109 (20.6)	257 (52.7)	47 (13.4)			
3+	18 (3.9)	34 (6.4)	40 (8.2)	258 (73.5)

**Table 3:** Concordance of HER2 status between CNB and SEB by clinical classification (n, %)

Core needle biopsy (CNB)	Surgical excision biopsy (SEB)			Concordance rate (%)	κ value (95%CI)	*p-*value
	Negative (n = 462)	Low (n = 1016)	Positive (n = 351)			
Negative	313 (67.7)	155 (15.3)	12 (3.4)	74.2	0.57	< 0.001
Low	131 (28.4)	787 (77.5)	81 (23.1)		(0.53-0.60)	
Positive	18 (3.9)	74 (7.3)	258 (73.5)			

Stratified by HER2 status in SEB (reference standard), the sensitivity and PPV of CNB were generally low, especially among IHC 1+ and 2+/ISH - groups, presenting the sensitivity 50.9%-52.7% and PPV 50.5%-55.2%; whereas, the specificity and NPV reached about 80% and more across all HER2 status (Table 4). When incorporating IHC 1+ and 2+/ISH - cases into HER2-low group; however, it showed the highest sensitivity (77.5%) and the lowest specificity (73.9%). This indicated that initial low sensitivities mainly resulted from the poor ability of CNB to distinguish between IHC 1+ and 2+/ISH - tumors, and the CNB was limited in accurately excluding HER2-low tumors.

**Table 4:** Sensitivity, specificity, PPV, and NPV of CNB in different HER2 statuses (%, 95%CI)

HER2 status in SEB	Sensitivity	Specificity	PPV	NPV
*Negative*				
0	67.7 [63.2–72.0]	87.8 [85.9–89.4]	65.2 [60.7–69.4]	89.0 [87.1–90.6]
*Low*	77.5 [74.7–80.0]	73.9 [70.7–76.9]	78.8 [76.1–81.2]	72.4 [69.2–75.4]
1+	50.9 [46.6–55.3]	79.7 [77.4–81.8]	50.5 [46.1–54.8]	80.0 [77.7–82.1]
2+/ISH -	52.7 [48.1–57.2]	84.4 [82.3–86.3]	55.2 [50.5–59.7]	83.1 [80.9–85.0]
*Positive*				
3+	73.5 [68.5–78.0]	93.8 [92.4–94.9]	73.7 [68.7–78.2]	93.7 [92.3–94.9]

*PPV* positive predictive value, *NPV* negative predictive value

### Factors associated with HER2 discordance between CNB and SEB

A total of 732 (40.0%) patients occurred discordance between CNB and SEB basing on pathological classification, while 471 (25.8%) basing on clinical classification and their distribution across variables of interest are shown in Table 5 and Table 6. The univariable and multivariable analyses showed that advanced N stages were inclined to be a stable indicative factor for the discordance of HER2 status by both pathological and clinical classification between these two tests (N1, N2-3 vs. N0 OR [95%CI] by univariable analysis: pathological discordance 1.34[1.08-1.67], 1.45[1.14-1.85]) and clinical discordance 1.27[0.99-1.63], 1.57[1.21-2.04]).

**Table 5:** Univariable and multivariable analyses of factors associated with the discordance of HER2 status by pathological classification, n (%)

Characteristics	Discordance	Univariable model	Multivariable model 1	Multivariable model 2	Multivariable model 3
	(n = 732)	OR [95% CI]	OR [95% CI]	OR [95% CI]	OR [95% CI]
*Age group (years)*					
≤ 35	56 (7.7)	1.00	1.00	–	–
36–45	215 (29.4)	1.04 [0.70–1.53]	1.05 [0.71–1.56]	–	–
46–55	227 (31.0)	0.84 [0.58–1.24]	0.87 [0.59–1.29]	–	–
56–65	148 (20.2)	0.83 [0.55–1.23]	0.85 [0.56–1.28]	–	–
> 65	86 (11.7)	0.89 [0.57–1.38]	0.91 [0.58–1.43]	–	–
*Tumor location*					
Unilateral	718 (98.1)	1.00	1.00	1.00	–
Bilateral	14 (1.9)	2.36 [1.02–5.48]	2.21 [0.95–5.17]	2.21 [0.95–5.15]	–
*Histological type*					
Others	49 (6.7)	1.00	1.00	1.00	–
Invasive ductal	683 (93.3)	0.71 [0.47–1.06]	0.75 [0.50–1.12]	0.73 [0.49–1.09]	–
*Histological grade*					
1	26 (3.6)	1.00	–	–	–
2	342 (46.7)	1.00 [0.60–1.69]	–	–	–
3	364 (49.7)	0.90 [0.54–1.52]	–	–	–
*pT stag*e					
T1	291 (39.8)	1.00	1.00	–	–
T2	367 (50.1)	0.90 [0.74–1.09]	0.86 [0.70–1.05]	–	–
T3–T4	74 (10.1)	1.14 [0.81–1.60]	0.99 [0.69–1.42]	–	–
*pN stage*					
N0	334 (45.6)	1.00	1.00	1.00	1.00
N1	223 (30.5)	1.34 [1.08–1.67]	1.33 [1.06–1.66]	1.34 [1.08–1.68]	1.35 [1.08–1.68]
N2–N3	175 (23.9)	1.45 [1.14–1.85]	1.42 [1.11–1.83]	1.43 [1.12–1.82]	1.45 [1.14–1.85]
*Type of surgery*					
Mastectomy	671 (91.7)	1.00	1.00	–	–
Lumpectomy	61 (8.3)	0.82 [0.59–1.13]	0.81 [0.58–1.14]	–	–
*ER status in SEB*					
Negative	180 (24.6)	1.00	–	–	–
Positive	552 (75.4)	1.29 [1.04–1.60]	–	–	–
*PR status in SEB*					
Negative	214 (29.2)	1.00	1.00	1.00	1.00
Positive	518 (70.8)	1.31 [1.07–1.60]	1.30 [1.06–1.60]	1.32 [1.08–1.61]	1.31 [1.07–1.61]

Model 1 is full model adjusting variables, including age, tumor location, histological type, pT stage, pN stage, type of surgery, and PR status in SEB. Model 2 is fitted by stepwise backward method adjusting variables, including tumor location, histological type, pN stage, and PR status in SEB. Model 3 is fitted adjusting variables pN stage and PR status in SEB, which showed statistically significant in univariable analysis (*P* < 0.05)

*ER* estrogen receptor, *PR* progesterone receptor, *SEB* surgical excision biopsy

**Table 6:** Univariable and multivariable analyses of factors associated with the discordance of HER2 status by clinical classification, n (%)

Characteristics	Discordance	Univariable model	Multivariable model 1	Multivariable model 2	Multivariable model 3
	(n = 471)	OR [95% CI]	OR [95% CI]	OR [95% CI]	OR [95% CI]
Age group (years)					
≤ 35	40 (8.5)	1.00	1.00	–	–
36–45	140 (29.7)	0.90 [0.59–1.37]	0.94 [0.62–1.45]	–	–
46–55	146 (31.0)	0.75 [0.50–1.14]	0.78 [0.52–1.20]	–	–
56–65	92 (19.5)	0.71 [0.46–1.10]	0.72 [0.46–1.13]	–	–
> 65	53 (11.3)	0.74 [0.46–1.21]	0.78 [0.48–1.28]	–	–
*Tumor location*					
Unilateral	460 (97.7)	1.00	1.00	1.00	1.00
Bilateral	11 (2.3)	2.68 [1.18–6.12]	2.46 [1.04–5.75]	2.43 [1.03–5.63]	2.43 [1.04–5.62]
*Histological type*					
Others	40 (8.5)	1.00	1.00	1.00	1.00
Invasive ductal	431 (91.5)	0.52 [0.34–0.78]	0.55 [0.36–0.84]	0.53 [0.35–0.81]	0.53 [0.35–0.82]
*Histological grade*					
1	15 (3.2)	1.00	–	–	–
2	195 (41.4)	0.99 [0.54–1.80]	–	–	–
3	261 (55.4)	1.23 [0.68–2.24]	–	–	–
*pT stage*					
T1	180 (38.2)	1.00	1.00	–	–
T2	238 (50.5)	0.98 [0.78–1.22]	0.90 [0.71–1.13]	–	–
T3–T4	53 (11.3)	1.36 [0.95–1.97]	1.03 [0.69–1.51]	–	–
*pN stage*					
N0	209 (44.4)	1.00	1.00	1.00	1.00
N1	140 (29.7)	1.27 [0.99–1.63]	1.24 [0.96–1.59]	1.26 [0.98–1.62]	1.27 [0.99–1.62]
N2–N3	122 (25.9)	1.57 [1.21–2.04]	1.50 [1.13–1.97]	1.53 [1.17–2.00]	1.53 [1.17–1.99]
*Type of surgery*					
Mastectomy	434 (92.1)	1.00	1.00	–	–
Lumpectomy	37 (7.9)	0.78 [0.53–1.14]	0.80 [0.53–1.17]	–	–
*ER status in SEB*					
Negative	145 (30.8)	1.00	–	–	–
Positive	326 (69.2)	0.81 [0.65–1.02]	–	–	–
*PR status in SEB*					
Negative	169 (35.9)	1.00	1.00	1.00	–
Positive	302 (64.1)	0.83 [0.66–1.03]	0.82 [0.66–1.03]	0.83 [0.66–1.03]	–

Model 1 is full model adjusting variables, including age, tumor location, histological type, pT stage, pN stage, type of surgery, and PR status in SEB. Model 2 is fitted by stepwise backward method adjusting variables, including tumor location, histological type, pN stage, and PR status in SEB. Model 3 is fitted adjusting variables tumor location, histological type and pN stage, which showed statistically significant in univariable analysis (*P* < 0.05)

*ER* estrogen receptor, *PR* progesterone receptor, *SEB* surgical excision biopsy

Since the histological grade and ER status were found significantly associated with PR status (chi-square test: *χ*^*2*^ = 892.8, 152.4, both *P* < 0.05), these two variables were not included in the following multivariable analysis to avoid collinearity. The multivariable model 1 including all potential influencing factors, model 2 developed by stepwise backward method, and model 3 adjusting only variables based on univariable analysis, all showed no obvious differences from those indicated by univariable analysis, with the area under the ROC curve of these three models being 0.58, 0.56, and 0.56 by pathological classification and 0.59, 0.58, and 0.57 by clinical classification, which suggested that there might be some important unobservable variables contributing to the discordance between CNB and SEB. In addition, it suggested that the PR-positive status and bilateral tumor location were associated with more discordance of HER2 status by pathological and clinical classification, respectively; while the invasive ductal cancer, compared with other histological type, was associated with less discordance of HER2 status by clinical classification.

## Discussion

Focusing on the newly proposed concept of HER2-low (IHC 1+ or 2+/ISH -) tumors, and verified effectiveness of trastuzumab deruxtecan (T-DXd) treating HER2-low breast cancer patients by a large RCT, our study addressed the diagnostic value of CNB in determining HER2 status, especially in HER2-low population, based on which, we found moderate concordances between CNB and SEB using either pathological or clinical classification (Concordance rate: 60% and 74%; Kappa value: 0.46 and 0.57); the specificity and NPV of CNB calculated according to three categories seemed not enough for accurately excluding HER2-low tumors, and the CNB might be poor to distinguish IHC 1+ and 2+/ISH - tumors. In addition, our study demonstrated the unique epidemiological characteristics of HER2-low patients comparing with those with HER2-negative or positive tumors and suggested the discordance between CNB and SEB might be more inclined to occur in patients with advanced N stages and some other specific tumor characteristics, but more potential influencing factors remained to be explored.

CNB, as a more convenient and less painful biopsy method, has been widely used in clinical diagnosis and molecular subtyping of breast cancer. Since it obtains tissue instead of merely cells, pathologists could perform IHC staining and other tests on these specimens and determine HER2 status as well as other biomarkers of lesions. Notably, CNB could play an important role in therapy decision-making for patients with advanced tumors or those who would receive neoadjuvant therapy, because surgical excision specimens could not be acquired in all these specific populations, the determinations of biomarkers can only rely on the results of CNB.

The accurate identification of HER2 status means much for the treatment and prognosis of breast cancer. Previously, anti-HER2 therapy can only be used for HER2-positive tumors, but current guidelines recommended such therapy can also be used for those with low HER2 status. Additionally, drugs targeting tumor cells with different HER2 statuses are not the same. HER2-positive tumors can be treated with monoclonal antibodies, tyrosine kinase inhibitors (TKI), antibody - drug conjugates (ADC), and others, while for HER2-low tumors, only T-DXd has proven to be effective [11]. Therefore, if CNB makes an incorrect judgment, it will result in loss of possible treatment, misuse of drugs, and unexpected side effects, thus leading to missing the best time for treatment and waste of medical resources.

The accuracy and reliability of CNBs have been reported in several studies. Meattini et al. reviewed several literatures and summarized the concordance of biomarkers (ER, PR, HER2, and Ki67) between CNB and SEB as 61.5-99.1% [21]. If we focus only on the HER2 state, the consistency varies greatly. In a comparative study of 209 breast cancer patients, the authors calculated HER2 concordances between CNB and SEB of only 56% (κ = 0.392) [17]. However, two other studies supported the reliability and accuracy of CNB in evaluating HER2 status, with a concordance rate of 96.3% (n = 298, κ = 0.894) and 84.8% (n = 1372, κ = 0.684), respectively [22, 23]. In our study, we analyzed the data and evaluated the concordance according to both the pathological and clinical classifications. The pathological classification is based on specialist consensus and guidelines, while the clinical classification is what most scholars used in their studies and what guides the anti-HER2 therapy nowadays. Our findings indicated a limited diagnostic value of CNB reaching an overall concordance rate of 60% (κ = 0.46) by pathological classification and 74.2% (κ = 0.57) by clinical classification, still relatively lower than previous similar studies basing on traditional classification. In our opinion, there exist no unified standards or definition for an appropriate diagnostic value and varied depending on trade-off about the consequences of misdiagnosis, cost of undergoing diagnosis, preferences, and values of clinicians and patients. Specifically for our study, the higher the consistency of CNB with SEB, the better the diagnostic value of CNB when setting SEB as the gold standard.

Specifically, HER2-low tumors might be misclassified as HER2-negative (15.3%) and HER2-positive (7.3%) tumors; HER2-negative (28.4%) and HER2-positive (23.1%) tumors might also be misclassified as HER2-low tumors by CNB. Besides, we investigated the validity of CNB at each IHC score level by SEB (reference standard). Interestingly, among IHC 1+ and 2+/ISH - subgroups, the sensitivity (50.9%-52.7%) and PPV (50.5%-55.2%) were especially low; however, in incorporated HER2-low group (IHC 1+ and 2+/ISH -), the sensitivity elevated while the specificity decreased. This indicated that the low sensitivity of CNB was caused mainly by the poor discrimination between IHC 1+ and 2+/ISH - tumors, although it might not affect treatment decision-making now because anti-HER2 therapy was recommended for both populations. It is noteworthy that the specificity and NPV of CNB in HER2-low tumors were low, which means the CNB is limited accurately excluding HER2-low tumors, thus quite a few HER2-negative tumors might be classified as HER2-low tumors. Incorrect determination may affect therapy decisions for patients, which can lead to improper treatment, severe adverse outcomes, and waste of medical resources. Therefore, caution should be exercised in the utilization of CNB in HER2-low tumors, especially with the increasing demand of effective treatment based on accurate diagnosis, and the current dilemma remains the need for a reliable and sensitive quantitative assay to verify the status of HER2 [24].

Several studies have provided traditional explanations about the discordance between HER2 statuses of CNB and SEB, which included the heterogeneity of tumors, delayed fixation in SEB, and peripheral sampling in CNB [25, 26]. In HER2-low tumors, the role of these factors may be more pronounced, thus affecting the pathological interpretation. Previous studies have shown that HER2-low tumors (especially those scored as IHC 2+) have stronger heterogeneity [27, 28], and HER2-low can be found in both hormone receptor-positive and triple-negative breast cancers [29]. Moreover, HER2-low tumors are more susceptible to poor specimen sampling and processing. One study reported that up to 85% of the patients who scored IHC 0 in local laboratories would be evaluated as IHC 1+ or 2+ in central laboratories, emphasizing the importance of high-quality and standard HER2 evaluations [30]. Variations in staining of HER2-low cases led by different antibodies and kits should also be considered. A global multicenter study used different assays to re-interpret IHC results in traditionally defined HER2-negative patients and reported a HER2-low concordance of 85.1% in Ventana 4B5 assay, while 78.2% in non-Ventana 4B5 assay [31]. In another study, the HER2 status was assessed by Ventana 4B5 and HercepTest antibodies, showing the detection rate of HER2-low tumors was 27.4% and 9.2%, respectively [32]. It hinted that Ventana 4B5 antibody may be more applicable for HER2-low detection, as well as helping developing better detection methods to identify patients with HER2-low tumors who may benefit from targeted therapy. Additionally, pathologists have considerable uncertainty in the interpretation of HER2-low cases, especially between HER2-low and HER2-negative tumors. A study reported that 15% pathologists were in dispute over whether HER2 was evaluated as IHC 1+ or 0 [33], while another study demonstrated a concordance of merely 26% between IHC 0 and IHC 1+ tumors among 18 pathologists [34].

Our study also aimed to explore the pathological and clinical characteristics that may influence the concordance of HER2 status between CNB and SEB. Although the univariable and multivariable analyses by both classifications showed results with small differences, we found that advanced N stages (i.e., lymph node involvement) might be a stable indicator for the discordance between CNB and SEB, while other factors still need further verification, and some potential important factors have not been explored. It is reported that in patients with positive lymph nodes, specific genes exhibited characteristic expression patterns were related to tumor heterogeneity [35], suggesting tumors with lymph node metastases tend to be more heterogeneous in their primary focus than those without axillary involvement. Thus, tumors with a higher N stage tend to feature stronger invasiveness and heterogeneity in their primary cancers, causing IHC discordance when tested at different times.

To solve problems regarding the limited diagnostic value of CNB and insufficient detection methods for determining HER2 status, especially in HER2-low tumors, we proposed several suggestions as follows. One is to improve the quality of CNB by standardized specimen sampling and processing, as well as precise slide interpretation by pathologists; if possible, it is better to confirm the results of HER2 status by SEB. For some special conditions, such as determining HER2 status among patients with advanced cancer or before neoadjuvant therapy, it is necessary to develop some new detection method, or improve the accuracy of CNB, or even IHC itself, by technological innovation or combination with other detection methods. Since low expression of HER2 based on mRNA and protein is common in all breast cancers [36], if we can determine the HER2 expression threshold of anticancer effects of T-DXd, more precise therapy decisions will be made. Whether the current HER2 detection methods are sensitive enough to accurately define the threshold or lower limit of HER2 expression in the beneficial population is an issue worth paying attention to in future.

This might be the first population-based study addressing the diagnostic value of CNB among HER2-low breast cancer patients, which is very important for clinical decision-making contributing to the effectiveness of treatment and prognosis of breast cancer patients. Besides, we used not only the concordance rate, kappa value as usual, but also calculated the sensitivity, specificity, positive, and negative predictive values across different HER2 statuses to comprehensively evaluate the agreement of CNB and SEB on both pathological and clinical classifications.

Our study has some limitations. First, this was a retrospective study in a single hospital, making it subject to selection bias and limited generalizable of such conclusion. Second, there may be variations in specific sampling operations and specimen processing in clinical work resulting from human, machine, or environment that could not be completely avoided. Third, the characteristics of breast cancer patients were not sufficient, limited by data availability in our HIS system, some important variables associated with the discordance of CNB and SEB might not be observed and determined. Therefore, our future work will focus on dealing with these critical issues by collecting more abundant information of extended, heterogeneous population through linking to other data sources or purpose-oriented survey, as well as conducting large, prospective, multicenter studies if possible. Besides, variations in specific sampling operations and specimen processing may exist but will not make a big influence with laboratory work conducted by the department of pathology in West China Hospital, which has been certificated by CAP (College of American Pathologists) and implements strict quality control supporting academic research.

## Data Availability

The datasets used and/or analyzed during the current study are available from the corresponding author on reasonable request.

## Abbreviations

HER2: Human epidermal growth factor receptor 2
ER: Estrogen receptor
PR: Progesterone receptor
IHC: Immunohistochemistry
ISH: In situ hybridization
T-DXd: Trastuzumab deruxtecan
CNB: Core needle biopsy
SEB: Surgical excision biopsy
NAST: Neoadjuvant systemic therapy
ISH: Fluorescence in situ hybridization
OR: Odds ratio
CI: Confidence interval
PPV: Positive predictive value
NPV: Negative predictive value
